# Using Light Microscopy and Liquid Chromatography Tandem Mass Spectrometry for Qualitative and Quantitative Control of a Combined Three-Herb Formulation in Different Preparations

**DOI:** 10.3390/molecules21121673

**Published:** 2016-12-06

**Authors:** Tun-Pin Hsueh, Wan-Ling Lin, Tung-Hu Tsai

**Affiliations:** 1 Institute of Traditional Medicine, School of Medicine, National Yang-Ming University, Taipei 11221, Taiwan; melaopin@gmail.com; 2 Department of Traditional Chinese Medicine, Kaohsiung Chang Gung Memorial Hospital and Chang Gung University College of Medicine, Kaohsiung 83301, Taiwan; 3 Department of Chinese Medicine, E-DA Hospital, I-Shou University, Kaohsiung 82445, Taiwan; slingr23@gmail.com; 4 Graduate Institute of Acupuncture Science, China Medical University, Taichung 404, Taiwan; 5 School of Pharmacy, College of Pharmacy, Kaohsiung Medical University, Kaohsiung 807, Taiwan; 6 Department of Chemical Engineering, National United University, Miaoli 36063, Taiwan

**Keywords:** Yin-Chen-Hao-Tang, mass spectrometry, scoparone, geniposide, rhein, herbal medicine

## Abstract

*Artemisia capillaries* Thunb, *Gardenia jasminoides* Ellis, and *Rheum officinale* Baill have been combined to treat jaundice for thousands of years. Studies have revealed that these herbs induce anti-hepatic fibrosis and anti-hepatic apoptosis and alleviate hepatic oxidative stress. This study aims to determine the quality and quantity of an herbal formulation (Chinese name: Yin-Chen-Hao-Tang) using physical and chemical examinations. Physical examination of Yin-Chen-Hao-Tang in pharmaceutical herbal products, raw fiber powders, and decoction preparations was performed using Congo red and iodine-potassium staining. A sensitive and validated method employing ultra-high-performance liquid chromatography tandem mass spectrometry (UHPLC-MS/MS) was developed to simultaneously quantify the bioactive compounds scoparone, geniposide, and rhein in the Yin-Chen-Hao-Tang formulation in different preparations. Physical examination indicated that cellulose fibers with irregular round shapes were present in the pharmaceutical herbal products. The developed UHPLC-MS/MS method showed good linearity and was well validated. The quantification results revealed that the decoction preparations had the highest amounts of geniposide and rhein. Scoparone appeared in pharmaceutical herbal products from two manufacturers. This experiment provides a qualitative and quantitative method using physical and chemical examinations to test different preparations of herbal products. The results provide a reference for clinical herbal product preparations and further pharmacokinetic research.

## 1. Introduction

The use of detection techniques for herbal medicines or phytomedicines has been increasingly studied in recent years. While therapeutic effects of medicinal plants have been discovered and utilized in the past, new potential compounds, ranging from medicinal herbs to new drugs, have been discovered and screened. To investigate the candidate compounds and the efficacy, safety, and quality of medicinal plants that contain hundreds of chemical constituents, separation and detection methods that enable rapid analysis of inherently complex herbs have been developed.

Various detection techniques, including ultraviolet detection (UV), fluorescence detection (FD), photodiode array detection (DAD), and mass spectrometry (MS), can offer excellent selectivity and sensitivity for known or unidentified structures in natural compounds. Liquid chromatography, coupled with mass spectrometry, is the most prevalent because it can collect data instantly from each chromatographic peak, even at low sample concentrations and with relatively short analysis times. In recent years, studies have shown that versatile high performance liquid chromatography (HPLC) coupled with mass spectrometry can be used to analyze complex natural products and metabolites. HPLC in combination with two or more MS experiments (tandem mass spectrometry, MS/MS) provides higher resolution to achieve better quantitative analysis and has become a routine analytical technique for the quantitative identification of herbal medicines.

Quantification analysis of bioactive compounds by LC-MS/MS has been applied to combined herbal medicines. For instance, twelve bioactive compounds were simultaneously determined in a Ge-Gen-Qin-Lian decoction using HPLC-MS/MS [1]. Ten flavonoids, four alkaloids and four saponins were separated from a Ban-Xia-Xie-Xin decoction under the developed methods of HPLC-MS/MS [2]. Six bioactive compounds, including rhein, that are found in *Rheum palmatum* L., the major herb of San-Huang-Xie-Xin-Tang, were quantified by HPLC-MS/MS for further pharmacokinetic study [3]. Analysis of the Ling-Gui-Zhu-Gan decoction revealed 90 tentatively identified compounds classified as flavonoids, coumarins, benzofurans, or coumestans by HPLC-hybrid electrospray ionization linear ion trap-Orbitrap mass spectrometry (HPLC-LTQ-Orbitrap-MS/MS) [4]. Bioactive components make qualitative control of herbal formulations possible due to this highly efficient technique to separate and identify constituents in complex medicinal herb materials.

*Artemisia capillaries* Thunb (Chinese herbal name, Yin-Cen-Hao), *Gardenia jasminoides* Ellis (Zhi-Zi), and *Rheum officinale* Baill (Da-Huang) were combined to prepare a formulation to treat jaundice. The formulation was named Yin-Chen-Hao-Tang (*Artemisia capillaries* decoction) and was first recorded in the book Shang-Han-Lun (Treatise on Cold Damage Diseases) two thousand years ago. The weight ratio of each herb component recorded in the book was 6:3:2 for *Artemisia capillaries* Thunb, *Gardenia jasminoides* Ellis, and *Rheum officinale* Baill. The therapeutic properties of this formulation have been demonstrated from its herbal composition. *Artemisia capillaries* Thunb has been experimentally verified to have anti-hepatic fibrotic, anti-inflammatory, and hepatoprotective activities due to its constituent bioactive compounds, such as scoparone (6,7-dimethylesculetin), β-sitosterol, capillarin, capillarisin, cirsimaritin, quercetin, and chlorogenic acid [5,6]. Another herbal component, *Gardenia jasminoides* Ellis, has anti-inflammatory, anti-angiogenic, and choleretic effects from its ingredients genipin and geniposide [7,8,9]. The known ingredient *Rheum officinale* Baill contains aloeemodin, rhein, emodin, gallic acid, and chrysophano-8-o-d-glucopyranoside. Several pharmacological effects, including hepatoprotective, nephroprotective, anti-inflammatory, anti-oxidant, anticancer, and antimicrobial activities, are derived from the biological activities of rhein and emodin [10,11,12,13,14,15]. The synergetic effects of 6,7-dimethylesculetin, capillarisin, chlorogenic acid, geniposide, and rhein, the major bioactive constituents of Yin-Chen-Hao-Tang, were reported to contribute to the therapeutic effects of the formulation [16,17]. Studies on three combinations of these herbal medicines have shown potent anti-hepatic fibrosis and anti-hepatic apoptosis activities and alleviated hepatic oxidative stress effects after oral administration of Yin-Chen-Hao-Tang in rats [18,19,20]. Literature reports have also demonstrated that aqueous extracts of Yin-Chen-Hao-Tang could diminish the infectivities of both herpes simplex virus 1 (HSV-1) and HSV-2 [21].

A large body of literature exists on the bioactive compounds of Yin-Chen-Hao-Tang and their synergetic therapeutic effects. However, quality control information for this commonly available herbal formulation is scarce. HPLC-MS/MS has provided rapid and efficient quantification analysis of bioactive compounds among various herbal formulations. Good quality control for herbal formulations entails not only thoroughly validated quantitative methods for HPLC-MS/MS analysis, as per bioanalytical international guidance, but also the physical characteristics of herbal product quality. Therefore, this study aimed to physically and chemically examine the herbal formula Yin-Chen-Hao-Tang in preparations of pharmaceutical herbal products, raw fiber powders, and a decoction by light microscopy inspection with Congo red staining, and to simultaneously quantitatively evaluate the bioactive contents of scoparone, geniposide, and rhein, providing full validation and quality control of this formulation.

## 2. Results

### 2.1. Optimization of LC-MS/MS Conditions

**Figure 1:**
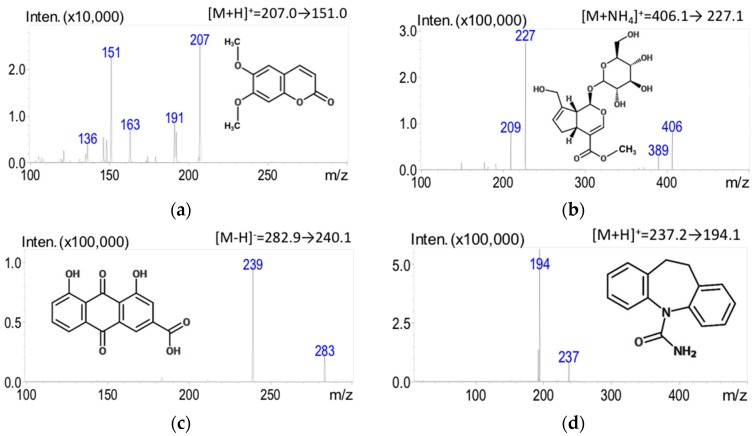
Chemical structures of the analytes and the product ion mass spectra and fragments of three marker compounds in YCHT and IS: (**a**) scoparone; (**b**) geniposide; (**c**) rhein; and (**d**) carbamazepine (IS).

The experimental LC-MS/MS spectra were acquired in the ESI (+) and ESI (−) ionization modes simultaneously. Initial optimization of the electrospray ionization mass spectrometer for the detection of bioactive compounds within the herbal formulation began with parent compound optimization. The optimizing step revealed large peaks at [M + H]^+^ for scoparone and carbamazepine (IS) at *m/z* 207.0 and 237.2 and at [M − H]^−^ for rhein at *m/z* 282.9. These peaks correlated with the expected molecular ion masses of the compounds. The optimized parent ion for geniposide was [M + NH_4_]^+^ at *m*/*z* 406.1 under the described mobile phase conditions. Eventually, the analytes in multiple-reaction monitoring (MRM) mode were found as the specific product ions for scoparone (*m/z* 207.0→151.0), geniposide (*m/z* 406.1→227.1), rhein (*m/z* 282.9→240.15), and carbamazepine (*m/z* 237.2→194.1) for quantitation (Figure 1). The collision energies used for quantitation are shown in Table 1.

**Figure 2:**
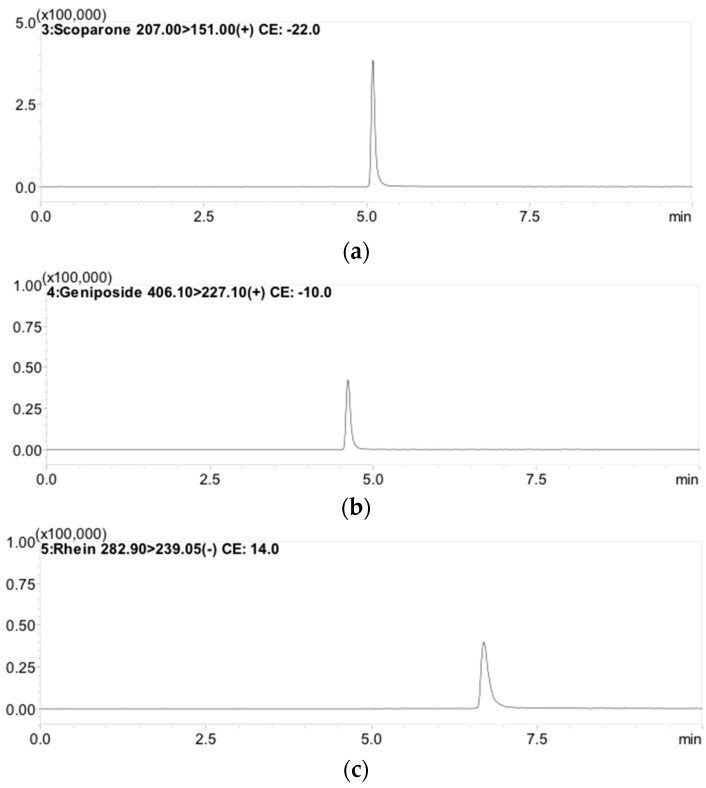
Typical MRM chromatograms of: (**a**) scoparone (RT: 5.3 min); (**b**) geniposide (RT: 4.7 min); and (**c**) rhein (RT: 6.9 min).

Optimization of LC-MS/MS conditions involved in analytical processes such as extracting solvent, mobile phase system, and gradient elution were essential. Several mobile phase combinations, such as acetonitrile-formic acid (0.1%) and methanol-water, were examined in different ratios to give a high-resolution peak in the chromatogram. As a result, methanol combined with 0.1% formic acid combined with 1 mM ammonium acetate buffer was selected as the solvent system. Good sensitivity, selectivity, and symmetrical chromatogram peaks with good resolution and co-elution after analysis and separation of multiple compounds by LC-MS/MS were achieved, despite the influence of gradient elution, column, and flow rate on the separation (Figure 2).

### 2.2. Method Validation

Each validation of the calibration curve contained three replicates of lower limit of quantification (LLOQ), low, mid, and high quality control standards (1, 10, 100, and 1000 ng/mL, respectively). Good linearity was obtained for the calibration curves, ranging from 1–100 ng/mL for scoparone, 5–1000 ng/mL for geniposide, and 5–500 ng/mL for rhein. The calibration curve equations were *y =* 11,829*x* + 43,450 for scoparone, *y* = 1931.5*x* + 4897.7 for geniposide, and *y* = 2776.9*x* + 6400.9 for rhein. The correlation coefficients (R^2^) used to evaluate the quality of the standard curves were above 0.995 for all analytical compounds in Yin-Chen-Hao-Tang. The limits of detection (LODs) are defined as three times the lowest concentrations of peak areas in chromatograms compared with the background signal-to-noise ratio. These limits also correspond to sensitivity: the lowest concentration can be reliably and reproducibly measured in at least three replicates. The LOD of scoparone, geniposide, and rhein by LC-MS/MS were 0.5, 1, and 1 ng/mL, respectively (Table 2).

The relative standard deviation (RSD) and bias values were used to determine the precision and accuracy of the quantitative analysis. The RSD values for intra-day and inter-day assays were within the ranges 1.69%–14.08% and 2.97%–12.93%, respectively. The accuracy ranged from –15.36%–9.83% for intra-day assays and from –9.34%–11.31% for inter-day assays. The analysis results revealed acceptable ranges of precision and accuracy for quantification and are summarized in Table 3.

### 2.3. Light Microscopy Photographs of Congo Red and Iodine Staining

**Figure 3:**
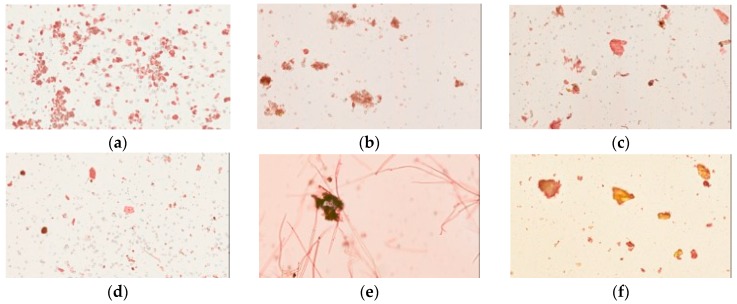

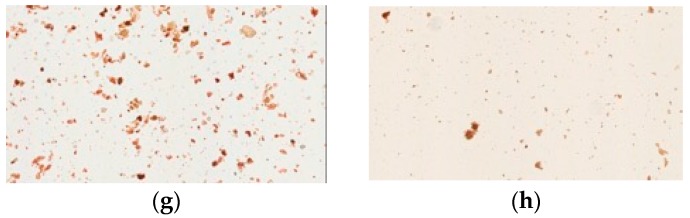
Light microscopy photographs of Congo red staining of Yin-Chen-Hao-Tang in preparations of pharmaceutical products: (**a**–**d**) products from brands A–D; raw herbal powders: (**e**) *Artemisia capillaries* Thunb; (**f**) *Gardenia jasminoides* Ellis; (**g**) *Rheum officinale* Baill; and (**h**) Yin-Chen-Hao-Tang decoction.

Congo red and iodine staining was used to identify cellulose fibers and cornstarch in the herbal pharmaceutical products. Congo red has strong affinity to cellulose fibers but not to their hydrolysis products; the pharmaceutical products were stained red or pink in photographs (Figure 3). These results revealed that samples of pharmaceutical products were red or pink under the light microscope, while the raw fiber powders of each herb in Yin-Chen-Hao-Tang were also stained red. However, the prepared Yin-Chen-Hao-Tang in the decoction demonstrated only sparse color.

**Figure 4:**
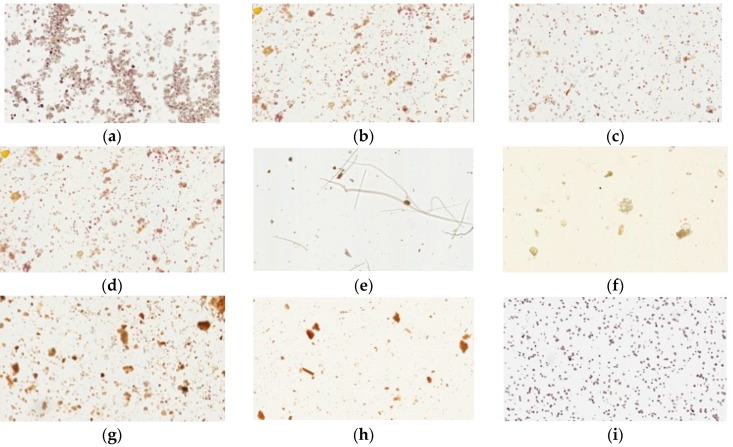
Light microscopy photographs of iodine staining of Yin-Chen-Hao-Tang in preparations of pharmaceutical products (**a**–**d**) products from brand A–D; raw herbal powders: (**e**) *Artemisia capillaries* Thunb; (**f**) *Gardenia jasminoides* Ellis; (**g**) *Rheum officinale* Baill; (**h**) Yin-Chen-Hao-Tang decoction; and (**i**) cornstarch.

Iodine forms an intense complex with glucose starch or glycogen to yield a blue color. Cornstarch, a source of glucose, is easily stained by iodine solution. Our physical examination showed that all samples of pharmaceutical products had dark blue or purple color under the light microscope, similar to cornstarch. In contrast, the raw fiber powders of each herb in Yin-Chen-Hao-Tang and the decoction gave no blue color (Figure 4).

### 2.4. Quantitative Determination of the Three Marker Compounds of YCHT Preparations

The aim of this experiment was to investigate the marker components of Yin-Chen-Hao-Tang from various commercially available pharmaceutical manufacturers. The essential component scoparone was only detected in pharmaceutical products from manufacturers A and B. In aqueous extracts, the components geniposide and rhein were measured at 0.840–8.972 mg/g and 0.040–0.093 mg/g among the commercial products, respectively. The extracted pharmaceutical powder in ethanol contained 0.984–1.968 mg/g geniposide and 0.043–0.187 mg/g rhein. Our experiment further quantified the decoction and the raw fiber powders of each herb in this formula as references. Analysis revealed that scoparone was not detected in either the raw fiber powder or the Yin-Chen-Hao-Tang decoction. Quantifications of geniposide in the raw fiber powders and the Yin-Chen-Hao-Tang decoction fell between 5.288 and 36.068 mg/g, while rhein quantification in the raw fiber powders and the decoction were between 0.0137 and 4.433 mg/g, respectively. All contents of marker components investigated for analysis are provided in Table 4*.*

## 3. Discussion

Yin-Chen-Hao-Tang has long been used for various liver diseases in clinical practice. In vivo studies have shown that administering Yin-Chen-Hao-Tang can reduce the concentrations of liver serum enzymes and potentially decrease collagen bundles thickening during the fibrosis process [22]. The formula, which consists only of three medicinal herbs, also induces anti-hepatic apoptosis and alleviates hepatic oxidative stress effects [19,20]. Concerning practical applications, therapeutic effects have been shown in animal experiments using Yin-Chen-Hao-Tang, and quality control standards have been created for this herbal formula.

Congo red and iodine staining was performed to examine the physical properties of the pharmaceutical products and the raw herbal powders. Congo red binds to cellulose molecules due to its symmetry and hydrogen bonding sites and, thus, is widely applied in phytochemistry to examine crude fibers [23]. Alignment between the diphenol backbone of Congo red and cellulose fibers imparts strong affinity to the binding site. Raw herbal powders of herbal medicines were filamentous and irregular in shape, and the samples that contained these cellulose fibers were stained red. The experimental results showed that all brands of pharmaceutical products exhibited granules that were multiform, with rod-like shapes in various distributions. In other words, compared to the raw powder fibers used as references, the final commercial pharmaceutical products contained added grinder-crushed herbal powder.

The iodine-potassium iodine staining experiment was performed to identify the starch contents in various brands of pharmaceutical products. Iodine can detect starch concentrations as low as 1 µg/mL and causes blue staining due to the starch’s amylose content [24]. The starch granule particles were blue and round compared to the fully stained cornstarch used as a positive control. The raw fiber powders were also stained with iodine but presented no blue color, indicating no starch content in the natural herbs or the decoction-prepared Yin-Chen-Hao-Tang. These results suggest that the prilling procedures likely involved additives that mixed and gelatinized during processing of the pharmaceutical products. A light microscopy study that physically inspected the pharmaceutical powders, the crude fibers and the decoction could provide further purity-indexed data to examine herbal formulations.

To quantify the amounts of ingredients in various commercially available pharmaceutical products and raw herbal powders, the most intense ion of each analyte detected in MRM mode was selected for quantitation. Under the described analytical conditions, scoparone, geniposide, and rhein underwent fragmentation of the parent ion to the product ion in the *m/z* ratios 207.0 to 151.0, 406.1 to 227.1, and 282.9 to 240.15, respectively. The selected LC-MS/MS mass ions used for determination of bioactive compounds in commercial pharmaceutical products were consistent with previous reports of scoparone, geniposide, and rhein [25,26,27]. The developed LC-MS/MS method for quantification of pharmaceutical YCHT products was well validated. All calibration curves obtained for scoparone, geniposide, and rhein exhibited good linear ranges from the LLOQ 10 ng/mL to 1000 ng/mL. The estimated R^2^ coefficients were all greater than 0.995, and all % RSD and % bias values were within 15%, indicating that the LC-MS/MS method provided excellent quantitative analysis of bioactive components in various preparations of Yin-Chen-Hao-Tang.

The quantification results demonstrate that the aqueous extract of geniposide, among the pharmaceutical powder samples from manufacturers A–C, ranged from 6.362 to 8.972 mg/g. However, geniposide in brand D was measured at only 0.840 mg/g, distinct from other manufacturers (Table 4). The raw powders of *Gardenia jasminoides* Ellis extracted in water contained approximately 5.288 mg/g geniposide, close to the values detected in the three pharmaceutical manufacturer products. In addition, the measured amount of geniposide was obviously increased in the extract raw fiber powder in ethanol, which corresponded with a previous study in which ethanol promoted the partition efficiency of geniposide [28,29]. However, the extracted amount of geniposide from pharmaceutical products in ethanol was equal to, or less than, the previously published levels, which may be due to the presence of additives during granulating process that decrease the partition capacity of geniposide.

Aqueous-extracted rhein from pharmaceutical manufacturers A and B was detected at 0.092 and 0.093 mg/g, whereas brands C and D had only half as much, at 0.040 and 0.045 mg/g. Compared to grinder-crushed crude powder from *Rheum officinale* Baill, the water-insoluble rhein was detected at only 0.014 mg/g, only one-fourth to one-tenth the levels in the other extracted pharmaceutical powders. However, the ethanol-extracted raw fiber powder of *Rheum officinale* Baill was detected at 0.996 mg/g, greater than other preparations of Yin-Chen-Hao-Tang. The synergic effects of multicomponent herbal medicines in a combined preparation affect the concentrations of bioactive compounds, distinct from a single preparation [30].

According to the quantitative results, although *Gardenia jasminoides* Ellis is not considered the major herb in this formula, geniposide was predominant among the three bioactive compounds. Geniposide has reported anti-inflammatory, anti-oxidant and hepatoprotective activities [31]. Rhein has anti-fibrotic, anti-inflammatory, anti-oxidant, and anti-tumorigenic effects [13,14,31]. Considering the synergetic effects of the three medicinal herbs in the Yin-Chen-Hao-Tang formulation, the prolonged potency of geniposide with rhein from the area under the curve (AUC) of pharmacokinetic parameters exhibited additive properties not observed with geniposide alone [32]. Based on the therapeutic properties of geniposide, it is supposed that the predominant geniposide, accompanied by rhein, plays an essential role in this formula.

In addition, there was no detectable scoparone in the herbal ingredients of any Yin-Chen-Hao-Tang preparation except the samples from manufacturers A and B. The quantitative results are not consistent with previous studies that identified scoparone in the decoction preparation. First, the organic properties of these compounds make dissolution of scoparone in water difficult. Second, the melting point of the standard 6,7-dimethoxycoumarin is 143–145 °C; thus, an ordinary process of Yin-Chen-Hao-Tang decoction would not easily include scoparone. Based on a review of previous studies on the pharmacokinetics and fingerprints of this formula, the preparation process includes dissolution of the freeze-dried Yin-Chen-Hao-Tang extraction powder with methanol [17,33], which simplifies disclosure of the bioactive component by analytical instruments. In addition, only the pharmaceutical products from brand A and B contained detectable scoparone, indicating that some crude fibers may not be fully isolated from the gelatinized pharmaceutical samples during the manufacturing processes.

Our quantification results also offer a geniposide-based translational amount of pharmaceutical product for Yin-Chen-Hao-Tang decoction. According to back-calculations, the 250 mL of prepared decoction contained 36.068 mg/g geniposide, which is approximately four times the content of geniposide in brand C and six-fold higher than the amount in brand A, while the rhein content in brand C was 1% of that in the Yin-Chen-Hao-Tang decoction. However, the amount of marker compound could be influenced by the processing methods for Chinese herbal medicine preparation, such as baking, decoction, frying, soaking, and granulation processes. Crude fiber powder extraction and additives used may also differ in the granulating processes among manufacturers. The experimental results for different preparations provide clinical references for pharmaceutical product dosing that are translational to the Yin-Chen-Hao-Tang decoction.

## 4. Materials and Methods

### 4.1. Reagents

All solvents, including LC/MS grade methanol (MeOH), ammonium acetate, and ethanol, were obtained from E. Merck (Darmstadt, Germany). Formic acid (98%–100%) was also purchased from E. Merck. Scoparone (6,7-dimethoxycoumarin) and rhein were both purchased from Sigma-Aldrich Research Biochemicals Inc. (St. Louis, MO, USA). Geniposide was obtained from Nacalai Tesque (Kyoto, Japan). The internal standard carbamazepine was provided from Research Biochemicals International Inc. (Natick, MA, USA). Triply deionized water was purified using a Q-Gard 1 Purification Cartridge water purification system from Millipore (Bedford, MA, USA) with a Millipak 40 Gamma Gold filter (0.22 µm) to produce particulate- and bacteria-free water. The pharmaceutical herbal products of Yin-Chen-Hao-Tang were purchased from Kaiser Pharmaceutical Co., Ltd. (Tainan, Taiwan), Sheng Chang Pharmaceutical Co., Ltd. (Taipei, Taiwan), Chuang Song-Zong Pharmaceutical Co., Ltd. (Kaohsiung, Taiwan), and Koda Pharmaceutical Co., Ltd. (Taoyung, Taiwan). Herbal medicines that comprise Yin-Chen-Hao-Tang, including *Artemisia capillaries* Thunb, *Gardenia jasminoides* Ellis, and *Rheum officinale* Baill, were prepared from Kaohsiung Chang Gung Memorial Hospital, Taiwan.

### 4.2. Instrumentation and Software

A Shimadzu UHPLC system consisting of a CBM-20A system controller, LC-20AD XR pumps, DGU-20A_3_ degasser, SIL-20AC XR auto sampler, and CTO-20A column oven coupled with an electrospray ionization (ESI) interface equipped with an LCMS-8030 triple quadrupole mass spectrometer (Shimadzu, Kyoto, Japan) were utilized for the separation and detection of bioactive compounds in Yin-Chen-Hao-Tang and carbamazepine (IS). The remote-controlled software for the Shimadzu UHPLC-MS/MS system (Kyoto, Japan) was LabSolutions v. 5.60 SP1. Statistical calculations were performed using Microsoft Excel.

### 4.3. High-Performance Liquid Chromatography Separation and Tandem Mass Spectrometry Detection

All samples were subjected to chromatographic separation using the Shimadzu HPLC system with an Acquity HPLC BEH C_18_ column (1.7 µm, 100 mm × 2.1 mm) (Waters Corp., Milford, MA, USA). Each sample was maintained in the autosampler at 4 °C; 10 µL of each sample was injected into the column. Chromatographic analyses were performed at 35 °C with a flow rate of 0.2 mL/min. The mobile phase consisted of 1 mM ammonium acetate with 0.1% formic acid in H_2_O (pH = 3) and methanol. A gradient elution was applied from 20% methanol (0–2 min) to 95% methanol and was held constant for 8 min (3–11 min) and decreased to 20% methanol at 12 min, with an overall run time of 20 min.

The MS/MS spectrometer operating conditions were optimized with a set interface voltage of 3.5 kV, a desolvation line temperature of 250 °C, heat block temperature of 400 °C, and a collision gas pressure of 230 kPa. The desolvation gas and drying gas was nitrogen, and the gas flow rates for the two conditions were 3 and 17 L/min, respectively. Argon gas was used for collision-induced dissociation (CID). All samples were detected using the MRM mode.

### 4.4. Stock and Working Solutions

The stock solutions of scoparone, geniposide, and rhein were prepared in 100% methanol at a concentration of 1 mg/mL. These stock solutions were diluted with 100% methanol to prepare a series of working solutions at 1, 5, 10, 50, 100, 500 and 1000 ng/mL for calibration curves. Quality control (QC) samples were prepared in the same manner at 5, 10, 50, and 100 ng/mL. All solutions were stored at −20 °C and were brought to room temperature for analysis.

### 4.5. Sample Preparation for Extracts of Pharmaceutical Products and the Decoction

Extract samples of 0.1 g of the above pharmaceutical products were immersed in 1 mL H_2_O or ethanol to a concentration of 100 mg/mL. After ultrasonication in a water bath for 10 min at room temperature, the samples were centrifuged at 13,000 rpm for 10 min at 4 °C. The supernatant was then collected and filtered through a 0.22-μm filter, and the filtrate was analyzed by LC-MS/MS at an appropriately diluted concentration. The Yin-Chen-Hao-Tang decoction was prepared as recorded in the original report, which consisted of boiling 22.5 g of *Artemisia capillaries* Thunb in 1 L of water for approximately 60 min until 500 mL water remained. *Gardenia jasminoides* Ellis (11.3 g) and *Rheum officinale* Baill (7.5 g) were added to the decoction for an additional 30 min to collect a final Yin-Chen-Hao-Tang decoction (250 mL).

### 4.6. Analytical Method Validation

The validation of all methods was based on published FDA bioanalytical method validation [34]. The LOD and the LLOQ were, respectively, defined as signal-to-noise ratios (S/N) of 3 and 10 for each standard. The standard calibration curve was used to determine the QC and unknown sample concentrations from the peak area ratios already obtained for these samples. The acceptance criteria for calibration curves were least-squares linear regression R^2^ values greater than 0.995. However, LLOQ was accepted within 20% of the nominal concentration, and all back-calculated values of the created standard calibration curves were required to be within 15%.

The intra-day precision was the coefficient of variation (%, CV) within a single day from six replicate analyses, and the inter-day precision was determined across all consecutive days. Accuracy was the deviation (%, bias) of estimated concentration and nominal concentration. The accuracy and precision were calculated as bias (%) = [(C_obs_ − C_nom_)/C_nom_] × 100% and RSD (%) = (standard deviation (SD)/C_obs_) × 100%. Accuracy and precision were required to be within ±15% of nominal values to be considered acceptable. The developed and validated method was applied to analyze four brands of commercial pharmaceutical herbal products, raw fiber powders, and a decoction. All samples were extracted and analyzed in triplicate.

### 4.7. Light Microscopy Photographs of Congo Red- and Iodine-Stained Samples

Samples were immersed in a mixture of glycerol and 20% ethanol (1:1) to make a 3% solution (*w*/*w*). One to two drops of the suspension were then added to a microslide, stained with 0.1% Congo red, and covered with a coverslip, avoiding bubbles. The iodine staining procedure was performed in the same manner, adding 10% iodine-potassium iodide solution instead of Congo red. Congo red and iodine staining were viewed by light microscopy (Olympus CKX41, Tokyo, Japan) under a total magnification of 100×, and photographs were taken.

## 5. Conclusions

In this study, a developed and validated LC-MS/MS method was used to determine the scoparone, geniposide, and rhein concentrations in various preparations of Yin-Chen-Hao-Tang simultaneously. The composition ratios of the Yin-Chen-Hao-Tang decoction were consistently labeled, but different amounts of the three marker ingredients in samples A–D from different manufacturers were present. The marker compound levels were complex and influenced by herbal processes, including growth time, storage, decoction method, granulation process, or product batch. The complexities of herbal medicine formulation, such as synergetic effects between herbs or analogues co-eluting during analysis, also increase the difficulties involved in quantification. Our data revealed detectable scoparone, geniposide, and rhein levels in samples from manufacturers A and B, which could be further selected to investigate the pharmacokinetics of these bioactive compounds. The quantitative results and the available data, despite the absence of scoparone values in some cases, which was not consistent with the previous research, were easily applied and similar under clinical administration.

## Figures and Tables

**Table 1:** Analytical LC-MS/MS conditions for the identification of the three marker constituents and carbamazepine.

Constituents	Molecular Weight	RT ^1^ (min)	Mass Fragments		Collision Energy (eV)
			Q1 Mass (amu)	Q3 Mass (amu)	
Scoparone	206.20	5.3	207.0 [M + H]^+^	151.0	−22
Geniposide	388.36	4.7	406.1 [M + NH_4_]^+^	227.1	−10
Rhein	284.22	6.9	282.9 [M − H]^−^	240.15	14
Carbamazepine (IS) ^2^	236.26	5.8	237.2 [M + H]^+^	194.1	−20

^1^ RT, retention time. ^2^ IS, Internal standard.

**Table 2:** Calibration curves, linear ranges, and detection limits of the three marker constituents by LC-MS/MS.

Constituents	Linear Range (ng/mL)	Calibration Curve	R^2^	LOD (ng/mL)
Scoparone	1–100	*y* = 15466*x* − 1167.7	0.996	0.5
Geniposide	5–1000	*y* = 2036.7*x* − 2543.7	0.999	1
Rhein	5–500	*y* = 3198.3*x* + 838.43	0.997	1

**Table 3:** Intra- and inter-day precision and accuracy for the determinations for the three compounds.

Nominal Conc. (ng/mL)	Intra-Day			Inter-Day		
	Observed Conc. (ng/mL)	Precision, RSD (%)	Accuracy, Bias (%)	Observed Conc. (ng/mL)	Precision, RSD (%)	Accuracy, Bias (%)
Scoparone						
1	1.02 ± 0.14	14.08	1.79	1.03 ± 0.13	12.40	3.12
5	5.02 ± 0.49	9.78	0.32	5.20 ± 0.64	12.31	3.94
10	10.08 ± 0.77	7.65	0.81	10.40 ± 1.04	10.02	4.02
50	50.94 ± 3.50	6.87	1.89	51.91 ± 4.45	8.57	3.82
100	99.39 ± 4.71	4.74	–0.61	98.12 ± 3.49	3.56	–1.88
Geniposide						
5	5.49 ± 0.25	4.53	9.83	5.57 ± 0.34	6.09	11.31
10	9.95 ± 0.46	4.61	–0.54	10.17 ± 0.55	5.45	1.73
50	48.91 ± 1.17	2.40	–2.18	49.93 ± 1.63	3.26	–0.13
100	101.32 ± 3.08	3.04	1.32	100.35 ± 2.98	2.97	0.35
500	501.13 ± 18.27	3.64	0.23	519.20 ± 25.51	4.91	3.84
1000	1090.58 ± 44.58	4.09	9.06	1107.45 ± 43.45	3.92	10.74
Rhein						
5	5.05 ± 0.43	8.43	0.95	4.53 ± 0.29	6.45	–9.34
10	9.39 ± 0.74	7.93	–6.10	9.92 ± 0.41	4.13	–0.84
50	50.57 ± 4.25	8.41	1.15	53.08 ± 6.22	11.72	6.17
100	99.17 ± 1.67	1.69	–0.83	103.37 ± 4.28	4.14	3.37
500	423.21 ± 9.03	2.13	–15.36	492.91 ± 63.74	12.93	–1.42

Data are expressed as the means ± standard deviations (*n* = 5).

**Table 4:** Quantitation of bioactive compounds from Yin-Chen-Hao-Tang in different brands of pharmaceutical herbal products and herbal crude extracts.

Compound	Scoparone (mg/g)		Geniposide (mg/g)		Rhein (mg/g)	
Brands	Water ^1^	Ethanol ^2^	Water ^1^	Ethanol ^2^	Water ^1^	Ethanol ^2^
A	0.268 ± 0.01	0.177 ± 0.09	6.362 ± 0.200	1.419 ± 0.120	0.092 ± 0.002	0.123 ± 0.003
B	0.207 ± 0.006	0.162 ± 0.041	7.241 ± 0.139	1.033 ± 0.254	0.093 ± 0.002	0.187 ± 0.049
C	ND	ND	8.972 ± 0.436	1.968 ± 0.414	0.040 ± 0.002	0.043 ±0.011
D	ND	ND	0.840 ± 0.046	0.984 ± 0.204	0.045 ± 0.002	0.135 ± 0.031
*Art.*	ND	ND	ND	ND	ND	ND
*Gar.*	ND	ND	5.288 ± 1.023	19.195 ± 2.461	ND	ND
*Rhe.*	ND	ND	ND	ND	0.0137 ± 0.006	0.996 ± 0.071
Decoction ^3^	ND		36.068 ± 0.553		4.416 ± 0.20	

Note: data are expressed as the means ± standard deviations (*n* = 3); ND, not detected; *Art.*, *Gar.*, and *Rhe*., represent the powders of *Artemisia capillaries* Thunb, *Gardenia jasminoides* Ellis, and *Rheum officinale* Baill, respectively. ^1^ Powders extracted in water. ^2^ Powders extracted in ethanol. ^3^ Decoction was prepared according to the original reported method.
